# A Five-Gene-Pair-Based Prognostic Signature for Predicting the Relapse Risk of Early Stage ER+ Breast Cancer

**DOI:** 10.3389/fgene.2020.566928

**Published:** 2020-10-29

**Authors:** Na Li, Hao Cai, Kai Song, You Guo, Qirui Liang, Jiahui Zhang, Rou Chen, Jing Li, Xianlong Wang, Zheng Guo

**Affiliations:** ^1^School of Medical and Information Engineering, Gannan Medical University, Ganzhou, China; ^2^Department of Bioinformatics, Fujian Key Laboratory of Medical Bioinformatics, Key Laboratory of Ministry of Education for Gastrointestinal Cancer, School of Basic Medical Sciences, Fujian Medical University, Fuzhou, China; ^3^Medical Big Data and Bioinformatics Research Center, First Affiliated Hospital of Gannan Medical University, Ganzhou, China; ^4^Department of Systems Biology, College of Bioinformatics Science and Technology, Harbin Medical University, Harbin, China; ^5^Key Laboratory of Arrhythmias of Ministry of Education, Shanghai East Hospital, Tongji University School of Medicine, Shanghai, China

**Keywords:** ER+ breast cancer, micro-metastasis, relapse risk, prognosis signature, gene expression

## Abstract

About 20–30% of early-stage breast cancer patients suffer relapses after surgery. To identify such high-risk patients, many signatures have been reported, but they lack robustness in data measured on different platforms. Here, we developed a signature which is robust across multiple profiling platforms, and identified reproducible omics features characterizing metastasis of estrogen receptor (ER)-positive breast cancer from the Gene Expression Omnibus database with the aid of the signature. Based on the stable within-sample relative expression orderings (REOs), we constructed a signature consisting of five gene pairs, named 5-GPS, whose REOs were significantly correlated with relapse-free survival using the univariate Cox regression model. Using 5-GPS, patients were classified into the low-risk and high-risk groups. Patients in the high-risk group have worse survival compared to those in the low-risk group using Kaplan-Meier curve analysis with the log-rank test. Applying 5-GPS to the RNA-sequencing data of stage I-IV breast cancer samples archived in The Cancer Genome Atlas (TCGA), we found that the proportion of the high-risk patients increases with the stage. The proposed REO-based signature shows potential in identifying early-stage ER+ breast cancer patients with high risk of relapse after surgery.

## Introduction

Breast cancer is the most common malignant cancer among women (Bao et al., 2019), and approximately 70% of breast cancer patients are estrogen receptor-positive (ER+) (Jemal et al., 2011; Cai et al., 2018; Mitobe et al., 2020). About 20–30% of early-stage breast cancer patients suffer a relapse after surgery, and these patients need adjuvant therapies to reduce the risk of relapse (Cardoso, 2003; Yamashita et al., 2020). The relapse after surgery mostly drives from lymph nodes metastasis (LNMs) or micro-metastases of preoperative tumor cells. Detecting LNMs, the sensitivity of current preoperative imaging techniques is only from 30.3 to 57.6%;most the small LNM (<1.0 cm in the greatest dimension) patients remain undetected (Huang et al., 2011). It is difficult to detect LNMs efficiently and accurately in routine examinations (Wang et al., 2014)and there exists a high rate of false-negative clinical reports for tiny lesions or micro-metastases (Li et al., 2016). Thus, there exists an urgent need to develop a prognostic signature to identify high-risk patients with micro-metastases and poor prognosis from early stage patients. These high-risk patients would be recommended adjuvant therapy.

Many prognostic signatures have been developed for predicting clinical outcome of breast cancer patients. For example, a 70-gene signature assay has been approved by the U.S. Food and Drug Administration for identifying breast cancer patients likely to develop distant metastases (van ’t Veer et al., 2002), as documented in the National Comprehensive Cancer Network Breast Cancer Clinical Practice guidelines 2020.V4 (please see the full guidelines on NCCN.org). However, the measurement of the 70-gene signature must be carried out at two central laboratories in the Netherlands and the United States (Beumer et al., 2016) in order to control the experimental batch effects, which limits its wide applications. Other prognostic signatures, based on risk scores, usually calculated as the sum of the weighted expression values of the signature genes (Sotiriou et al., 2006; Wang et al., 2006). This type of signatures usually have low reproducibility across laboratories and platforms due to that there are large variations in the absolute gene expression values profiled by current RNA-sequencing (RNA-Seq) and microarray techniques (Seqc Maqc-Iii Consortium, 2014). Additionally, the gene expression measurements are also greatly affected by the sampling locations which may lead to varied proportions of tumor epithelial cells (Xu et al., 2015; Cheng et al., 2017) and RNA degradation problem during sample preparation (Chen et al., 2017).

In the contrast, our previous study has proved that the signatures based on the within-sample relative expression orderings (REOs) are highly robust against experimental batch effects, obviating the requirement of data normalization (Guan et al., 2016; Qi et al., 2016). Besides, the most important feature of the REO-based signature is that it is relatively robust against proportion variations in tumor epithelial cells due to the uncertainty of sampling locations (Xu et al., 2015; Cheng et al., 2017) and against certain RNA degradation during sample preparation (Chen et al., 2017). Cai et al. (2015) constructed a REO-based prognostic signature for early stage ER+ breast cancer. However, the signature was not validated in the data cohorts measured by platforms other than the Affymetrix platform. Therefore, it is worth adopting the REO-based approach to develop robust prognostic signatures.

In this study, we developed a signature for identifying early-stage ER+ breast cancer patients with high risk of relapse after surgery, which can aid the diagnosis of occult metastasis of early-stage breast cancer. Applying the signature in ER+ stage I-IV breast cancer samples from The Cancer Genome Atlas (TCGA), the proportion of the high-risk samples in each stage increase with the stage level. Based on the reclassified metastasis status of breast cancer samples of TCGA, we identified genomic features characterizing metastatic tissues.

## Materials and Methods

### Data Acquisition and Pre-processing

The breast cancer gene expression datasets analyzed in this study were downloaded from the Gene Expression Omnibus (GEO^1^) (Barrett et al., 2013) in August 2016, the European Genome-Phenome Archive (EGA^2^) in October 2016 with authorization (Lappalainen et al., 2015) and The Cancer Genome Atlas (TCGA^3^) (International Cancer Genome Consortium et al., 2010) in November 2017, as described briefly in Table 1. The detailed clinical information of all the datasets is given in Supplementary Table S1.

**TABLE 1:** Description of ER+ breast cancer tissue datasets used in this study.

		**Dataset**	**Platform**	**Metastasis group**	**Non- metastasis group**
Discovery cohort	Significantly stable gene pairs	GSE19615	Affymetrix GPL570	21	19
		GSE43365	Affymetrix GPL570	6	52
		GSE31448	Affymetrix GPL570	78	30
		EGAS00000000083	Illumina GPL6947	99	184
		Dataset	Platform		LN-
	Prognosis gene pairs	GSE7390	Affymetrix GPL96	/	134
		GSE6532	Affymetrix GPL96	/	85
Validation cohort	Validation	GSE2034	Affymetrix GPL96	/	209
		GSE4922	Affymetrix GPL96	/	116
TCGA data		Stage I	Stage II	Stage III	Stage IV
		126	350	142	15

*Note: / represents there have no such samples.*

In this study, the lymph node-positive samples with 4 or more node, stage T2–T4 and the tumor size above 20 mm were defined as true metastasis samples and the lymph node-negative samples with 0 node, stage T1 and tumor size of 20 mm or less were defined as non-metastasis samples, respectively. The ER+ breast cancer patients from GSE7390, GSE6532, GSE2034, and GSE4922 did not receive any adjuvant treatment after surgery. The clinical characteristics for discovery and validation cohort were summarized in Supplementary Table S2.

For the data measured by the Affymetrix’s microarray platform, we downloaded the raw data (.CEL files) and used the Robust Multi-array Average algorithm (Irizarry et al., 2003) for background adjustment without quantize normalization. Each probe-set ID was mapped to its Entrez gene ID with the corresponding CDF files. If a probe was mapped to multiple or zero genes, the data of this probe was dropped. If multiple probes were mapped to one gene, the expression value for the gene was summarized as the arithmetic mean of the values of the probes. The number of gene matched in GPL570 is 20486, 12752 in GPL96, 18926 in the TCGA microarray datasets and 12273 genes matched the EGA microarray datasets. The number of common genes shared by all datasets is 11792. The percentage of dropped genes was 62.53 and 42.77% in the expression data measured by GPL570 and GPL96. While 68.71 and 65.21% were dropped form the TCGA and EGA dataset. The probe id, Entrez ID and matched genes were listed in Supplementary Table S3.

For the data measured by the Illumina’s microarray platform, we directly downloaded the processed data. For TCGA datasets, the integrated data including both level-3 mRNA profiles and level-2 gene mutation profiles were obtained from the TCGA portal, and level-4 copy number data were downloaded from Firehose^4^.

### Identification of Significantly Stable REOs

**FIGURE 1:**
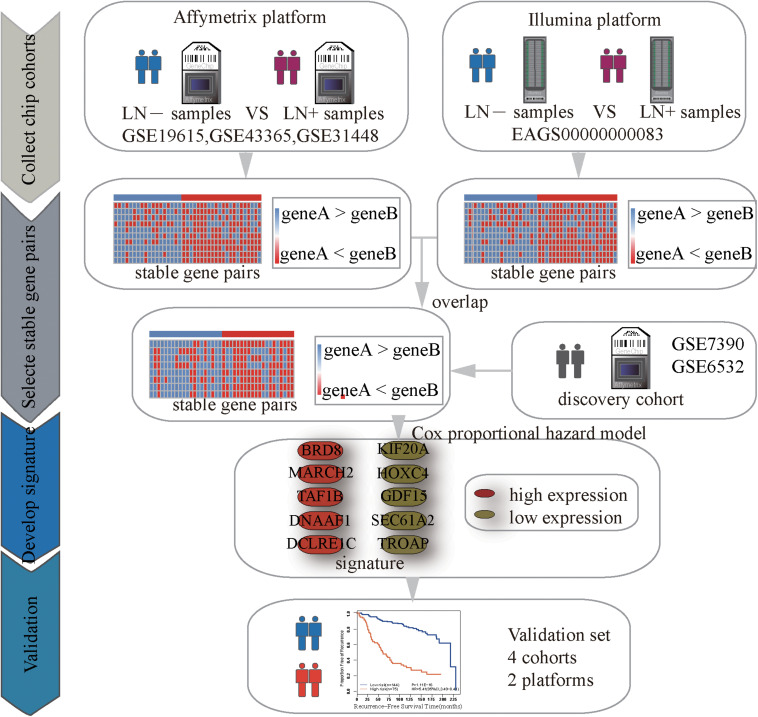
Flowchart of the processes for developing and validating the prognosis signature.

Figure 1 describes the process of developing and validating the prognosis predictor. The REO of two genes, A and B, is denoted as A > B (or A < B) if gene A has a higher (or lower) expression level than gene B. The stable significance of a REO is determined by a binomial distribution (Bahn, 1969) as follows:

$$p=\sum_{i=0}^{k-1}\left(\begin{array}{c} n\\ i \end{array}\right)\,p_{0}^{i}\,{\left(1-p_{0}\right)}^{n-i}$$

where *n* denotes the total number of samples in one dataset, *k* denotes the number of samples that have a certain REO pattern (e.g., A > B or A < B) in *n* samples and *p*_0_ (=1/2) is the probability of observing a certain REO pattern in a sample by chance. In other words, a pair of genes, A and B, is considered as statistically stable if the same order (A < B or A > B) is held in most of the samples with the binomial distribution is used to calculate the *p* value under the null hypothesis (A and B does not have a stable order relation) for the large-scale samples. If a gene pair have a statistically stable REO in both the non-metastasis group and metastasis group, respectively, but a reverse direction (A > B in one group but A < B in the other group), they form a REO reversal gene pair. The *p*-values were adjusted using the Benjamini-Hochberg method (Benjamini and Hochberg, 1995) for multiple tests.

### Algorithm for Searching the Optimal Signature

For a set of gene pairs whose REOs were associated with the relapse-free survival (RFS), a forward-stepwise selection algorithm was performed to search for an optimal subset of these gene pairs with the highest maximum concordance index (C-index). One gene pair with the largest C-index as the seed signature, candidate gene pairs were added to the signature one at a time until the further addition did not improve the predictive performance.

### Survival Analysis

The univariate Cox proportional hazards regression model was used to evaluate whether a REO reversal is significantly associated with the relapse risk of the patients. For each gene pair (Gene A, Gene B) in this study, the REO pattern of non-metastasis samples groups is that the expression level of Gene A is higher than that of Gene B (Gene A > Gene B), and REO reversal of metastasis samples groups means that the expression level of Gene A is lower than that of gene B (Gene A < Gene B). The independent prognostic value of a signature was assessed by multivariate Cox proportional hazards regression model after adjusting for clinical factors including age, grade and tumor size. The concordance index (C-index) proposed by Harrell et al. (1996) was used to evaluate the overall concordance between the risk classification and the observed RFS time with the “survival” R package. C-index, ranging from 0.5 (indicating random chance) to 1 (indicating prefect discrimination), is one of the most appropriate indexes for studies focusing on risk prediction. Survival curves of RFS between different groups were estimated with the Kaplan-Meier method and the *p*-value for the difference between the survival curves was calculated by the log-rank test (Harrington and Fleming, 1982) and drawn with the “ggplot2” R package. The predictive accuracy of the signature was assessed using the time-dependent, receiver operating characteristic curve (ROC) (Heagerty et al., 2000) with the “survivalROC” R package and AUC (the area under the ROC curve) was calculated. Time point of the ROC curve was set as 60 months. All statistical analyses were performed using the R software package version 3.2.0.

### Genomic Data Analyses

Fisher’s exact test was used to detect genes which had significantly different mutation frequencies or CNA frequencies between two groups classified by the prognostic signature. Spearman rank correlation analysis was used to estimate the correlation of genes expression levels with the CNAs. Significance level was defined as *p* < 0.05 or FDR < 0.05 for multiple testing.

## Results

### Development of Prognostic Signature for Postoperative Relapse Risk

Using the gene expression profiles of ER+ breast cancer samples, integrated from three datasets (GSE19615, GSE43365, GSE31448) measured by the Affymetrix’s microarray platform (Table 1 and Supplementary Table S3), we identified 1,442,839 significantly stable gene pairs, each of which had a stable REO in the 105 metastasis samples and a reversely stable REO in the 101 non-metastasis samples (Binomial test, FDR < 0.05). Similarly, using the data measured by the Illumina’s microarray platform from the European Genome-Phenome Archive, we identified 79,732 gene pairs with significantly stable REOs in the 99 metastasis samples and reversely significantly stable REOs in the 184 non-metastasis samples (FDR < 0.05, binomial test). The two lists of gene pairs shared 1690 common pairs (Supplementary Table S4) with consistent REO patterns, defined as the metastasis-related gene pairs.

The 219 samples of lymph node negative patients accepting surgery only, collected from the GSE7390 and GSE6532 datasets measured by Affymetrix, were used as the discovery cohort to develop a prognostic signature of postoperative relapse risk. From the 1690 metastasis-related gene pairs, using the univariate Cox proportional hazard model, we identified 71 gene pairs whose REOs were significantly (FDR < 0.05) associated with the RFS time (Supplementary Table S5). Here, RFS was used in a broad sense to represent the prognostic end points of both local relapse and distant relapse (Hudis et al., 2007; Suciu et al., 2018). Then, a forward-stepwise selection algorithm was performed to obtain a subset of gene pairs with the maximum C-index. Five gene pairs (Table 2) were obtained as the signature, denoted as 5-GPS, to predict the postoperative relapse risk based on the majority rule. In train data, we have found the non-metastasis samples (low-risk) with REO pattern of Gene A > Gene B and metastasis samples (high-risk) with REO pattern of Gene A < Gene B in Table 2. If gene B have a higher expression level than gene A in 3 or more pairs among 5 gene pairs tabulated in Table 2, the sample was classified as relapse high-risk, otherwise low-risk.

**TABLE 2:** The genes information of 5-GPS.

**Gene A**			**Gene B**		
**Gene ID**	**Official symbol**	**Official full name**	**Gene ID**	**Official symbol**	**Official full name**
10902	BRD8	bromodomain containing 8	10112	KIF20A	kinesin family member 20A
51257	MARCH2	membrane associated ring-CH-type finger 2	3221	HOXC4	homeobox C4
9014	TAF1B	TATA-box binding protein associated factor, RNA polymerase I subunit B	9518	GDF15	growth differentiation factor 15
123872	DNAAF1	dynein axonemal assembly factor 1	55176	SEC61A2	SEC61 translocon subunit alpha 2
64421	DCLRE1C	DNA cross-link repair 1C	10024	TROAP	trophinin associated protein

*Gene A has a higher expression level than Gene B in non-metastatic breast tissues.*

**FIGURE 2:**
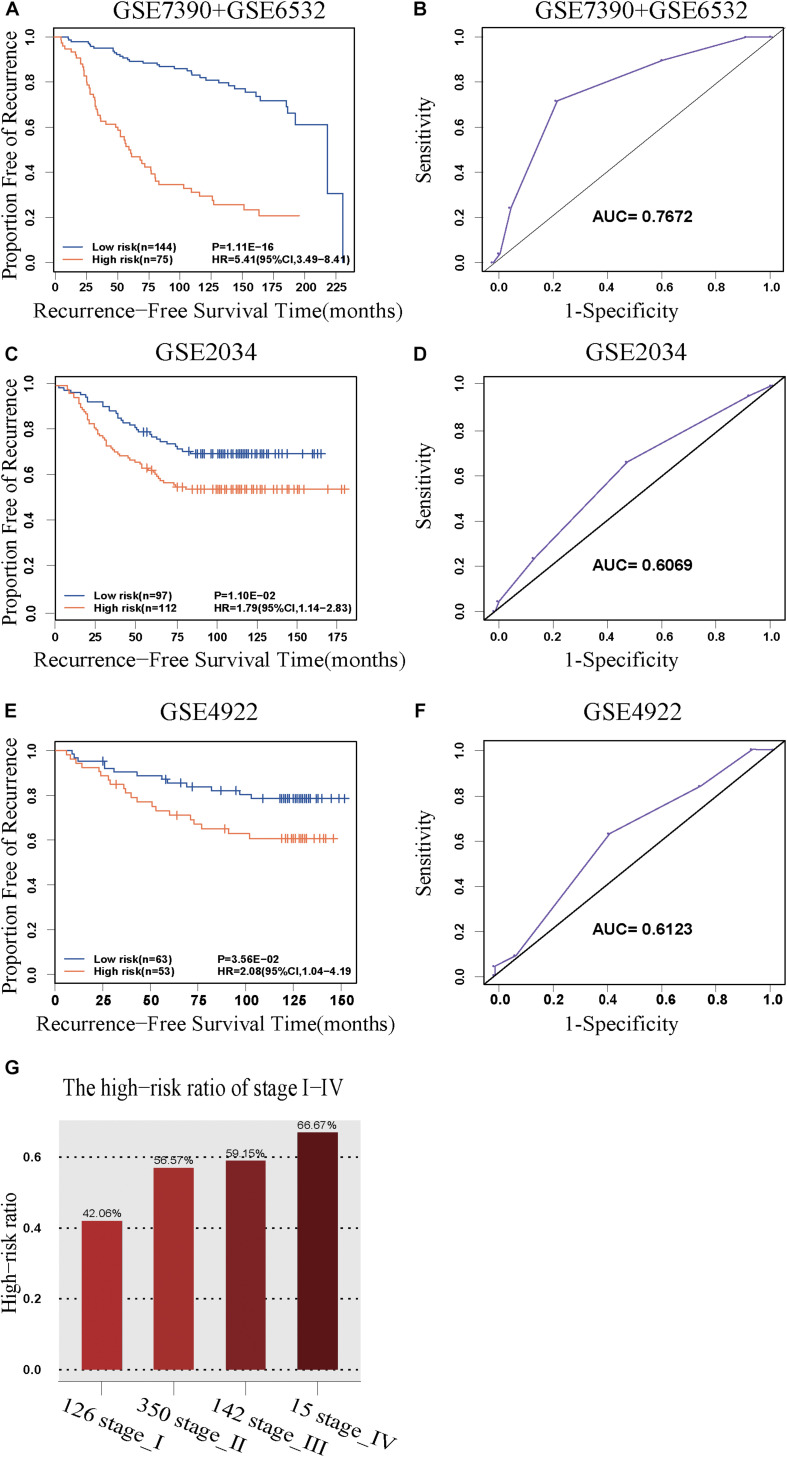
The predictive performance of the 5-GPS signature. The Kaplan-Meier curves of RFS for the early-stage breast cancer patients accepting surgery only in **(A)** the discovery cohort and **(C,E)** the validation cohorts. **(G)** The proportion of high-risk samples in I-IV stage. **(B,D,F)** The ROC curves for 5-GPS.

The discovery cohort was classified by 5-GPS into a low-risk group with 144 patients and a high-risk group with 75 patients (Supplementary Table S6). As shown in Figure 2A, the patients in the low-risk group had a significantly better RFS than those in the high-risk group (Hazard ratio (HR) = 5.41, 95% confidence interval (CI): 3.49–8.41, *p* = 1.11E-16, C-index = 0.71). The AUC was 0.7672 (Figure 2B).

### Validation of the 5-GPS Signature

The 5-GPS signature was firstly validated in two independent microarray datasets (Supplementary Table S6). In the GSE2034 dataset, the 5-GPS identified 97 patients with low risk of relapse and 112 patients with high risk of relapse, and the RFS of the former were significantly better than that of the latter (HR = 1.79, 95%CI: 1.14–2.83, *p* = 1.10E-02, C-index = 0.54, Figure 2C). The AUC was 0.6069 (Figure 2D). In the GSE4922 dataset, the low-risk group of 63 patients identified by 5-GPS had a significantly better RFS than the high-risk group of 53 patients (HR = 2.08, 95%CI: 1.04–4.19, *p* = 3.56E-02, C-index = 0.59, Figure 2E). The AUC was 0.6123 (Figure 2F). As expected, we also found the similar difference in RFS between low-risk and high-risk patients identified from integrated data of GSE2034 and GSE4922 (Supplementary Figure S1A) and integrated data of GSE7390, GSE6532, GSE2034 and GSE4922 (Supplementary Figure S1B). In the Multivariate Cox analyses for the discovery and validation cohorts all showed the prognostic signature was a strong independent factor for predicting the postoperative relapse risk after adjusting age, tumor size and histology grade (Table 3 and Supplementary Figure S2).

**TABLE 3:** Univariate and multivariate Cox regression analysis.

	**Multivariate model**		**Univariate model**	
**Variables**	**HR (95%CI)**	***p***	**HR (95%CI)**	***p***
**The 201 samples of the discovery cohort**				
5-GPS	4.89 (3.05–7.86)	4.99E-11	5.00(3.16–7.88)	3.09E-14
Age (>55 vs. < 55)	0.99(0.98–1.00)	0.16	1.00(0.99–1.01)	0.53
Grade (3 vs. 2 vs. 1)	0.85(0.57–1.27)	0.43	1.24(0.85–1.80)	0.27
Size (>2 vs. < 2 cm)	1.35 (1.00–1.83)	0.05	1.22(0.96–1.55)	0.10
**The 116 samples of the validation cohort**				
5-GPS	1.98 (0.96–4.08)	0.06	2.08(1.04–4.19)	0.04
Age (>55 vs. < 55)	0.90(0.42–1.92)	0.78	0.97(0.46–2.04)	0.93
Grade (3 vs. 2 vs. 1)	1.61 (0.91–2.84)	0.10	1.73(1.00–3.01)	0.05
Size (>2 vs. < 2 cm)	2.20(1.08–4.47)	0.03	2. 70(1.36–5.36)	0.003

Then we further tested 5-GPS on the RNA-Seq data from TCGA (Supplementary Table S6). After screening both accepting surgery and having follow-up data, only 7 ER+ breast cancer patients who received surgery but without further adjuvant therapy were left. Survival analysis in these patients cannot be performed. Endocrine therapy, chemotherapy and radiotherapy all have a great effect on prognosis of breast cancer. The significantly different survival between populations with irregular medical meta information cannot prove the good performance of 5-GPS in TCGA due to the effect of treatment on survival. In consideration of a strong correlation between stage level and RFS, we analyzed the proportion of high-risk samples identified by the signature in each stage rather than carrying out the survival analysis to test 5-GPS on the RNA-Seq data of 633 female ER+ stage I-IV breast cancer samples from TCGA. From Figure 2G, we see that the proportion of the high-risk samples in each stage increase with the stage level. The conformity implies the application feasibility of 5-GPS in the RNA-Seq data though it was obtained from the microarray data, supporting the cross-platform robustness of 5-GPS. We also compared the clinical information of AJCC metastasis pathologic of the high-risk and low-risk patients classified by 5-GPS. Among the 633 female ER+ stage I-IV breast cancer samples from TCGA, there are 15 patients with pathologic M1 and 903 patients with pathologic M0 (Supplementary Table S1). The 5-GPS classified 330 M0 patients into high-risk group, indicating lymphatic metastasis. And five M1 patients were stratified into low-risk group, where tumor cells may metastasize by other modes like hematogenous metastasis.

The expression profiles are dependent on the proportion of tumor epithelial cells in clinical samples. Our previous study showed that the REO-based signature is relatively robust against proportion variations of tumor epithelial cells (Cheng et al., 2017). Taking advantage of proportions of tumor epithelial cells in breast cancer samples from TCGA, we further confirmed the conclusion. The 633 samples were divided into a high-purity group with more than 50% tumor epithelial cells and a low-purity group with less than 50% tumor epithelial cells. Detailed information is given in Supplementary Table S1. The growth trend of proportions of the high-risk samples identified by 5-GPS was found in both the low-purity group (Supplementary Figure S3A) and the high-purity group (Supplementary Figure S3B). When the low-purity and high-purity group were classified by 60% tumor epithelial cells, similar increase were found (Supplementary Figures S3C,D). The line chart in Supplementary Figure S2 represents the number of samples in each stage.

Taken together, the above results demonstrated that the 5-GPS could robustly predict the relapse risk of ER+ breast cancer patients using samples profiled by microarray or RNA-Seq platforms. For each breast cancer sample, the prediction result of each signature gene pair was summarized in the Supplementary Table S6. In the above datasets measured by the Affymetrix platform, 5-GPS performed comparably with 9-GPS, a prognostic signature previously constructed by Cai et al. (2015) (Supplementary Figure S4 and Supplementary Table S7). However, 9-GPS performed poorly in the RNA-Seq data of TCGA, where the proportion of high-risk samples was at least 95% in every stage and sustained a weak increase with the stage level (Supplementary Figure S5). We used the hybrid model, combination of 5-GPS and 9-GPS, to validate the datasets. If the results of 5-GPS and 9-GPS are consistent, it is considered to be the high- and low-risk group, otherwise it is the difference group (Supplementary Table S7 and Supplementary Figure S6). From the results, the hybrid model performs better than the single model.

### Genomic Characteristics of the Prognostic Groups

We further investigated genomic differences between the two prognostic groups of the TCGA samples. Among the 633 samples with transcriptional data, 519 samples also have somatic mutation data and 622 samples have copy number aberrations (CNAs) data. This allowed us to further characterize the two prognostic groups with genomic profiles.

**FIGURE 3:**
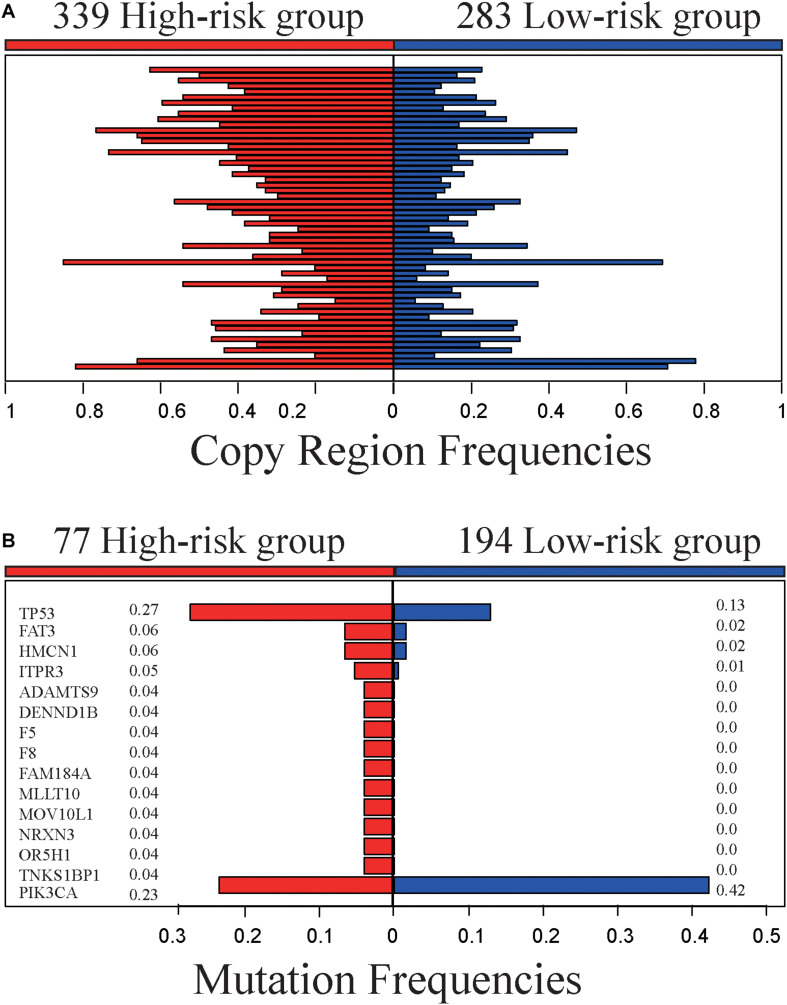
Genomic characteristics of the high- and low-risk groups. **(A)** The copy region frequencies of high- and low-risk group. **(B)** The mutation frequencies of high- and low-risk group.

The 622 samples with copy number alteration data were stratified into 339 high-risk and 283 low-risk samples by 5-GPS. Comparing 156 stage III-IV samples with 466 stage I-II sample, there were only five chromosome regions with significantly different frequencies of amplification (1 region) or deletion (4 regions) (Fisher’s exact test, FDR < 0.05). However, we found 55 chromosome regions with significantly different frequencies of amplification (20 regions) or deletion (35 regions) (Figure 3A and Supplementary Table S8) between the 339 high-risk samples and the 283 low-risk samples (Fisher’s exact test, FDR < 0.05). The high-risk group include 94 stage III-IV patients and the low-risk group include 221 stage I-II samples. In addition, 54 of the 55 regions had higher aberration frequencies in the high-risk group than in the low-risk group, providing further evidence for the higher degree of instability in the genomes of the high-risk patients. Within the 55 chromosome regions, the expression levels of 35 genes were significantly correlated, positively, with their CNA frequencies (Spearman correlation, *p* < 0.05). Many genes in these chromosome regions, such like *ERLIN2* (amp 8p11.23) (Wang et al., 2012), *VAV2* (del 9q34.2) (Tan et al., 2017), *ADAMTS6* (del 5q12.3) (Xie et al., 2016)and *NCS1* (del 9q34.11) (Moore et al., 2017), are known to be related with tumor invasion and metastasis.

The 519 samples with somatic mutation data were stratified into 277 high-risk samples and 242 low-risk samples by 5-GPS. In the high-risk group, 77 are stage III-IV patients and 200 are stage I-II patients. In the low-risk group, 194 are stage I-II patients and 48 are stage III-IV patients. We compared 77 stage III-IV high-risk patients and 194 stage I-II low-risk patients and identified 15 genes that had significantly different mutation frequencies (Fisher’s exact test, *p* < 0.05). Furthermore, 14 of 15 genes had significantly higher mutation rates in the high-risk group than in the low-risk group (Figure 3B and Supplementary Table S9), suggesting that the high-risk samples had an increased degree of genomic instability. For example, *TP53* mutated in 27.3% of the 77 high-risk samples but only in 12.9% of the 194 low-risk samples. It is well known that *TP53* aberrations could induce genomic instability, aggravate tumor progression and promote tumor metastases (Marchetti et al., 1993; Reichel et al., 1994; Haase et al., 2019). *PIK3CA* had lower mutation frequencies in the high-risk patients than in the low-risk patients. *PIK3CA* mutations may be associated with worse clinical outcomes (Mollon et al., 2019). In comparison, 63 genes with significant mutation frequencies were identified by comparing all stage III-IV samples with all stage I-II samples using Fisher’s exact test (*p* < 0.05) and the mutation frequencies are low. Only one gene, *FAT3*, mutated in more than 5% stage III-IV cancer samples, and there is no report in literature to our knowledge on this gene’s relation with breast cancer relapses and metastasis.

Taken together, the high-risk samples predicted by 5-GPS are characterized with distinct genomic lesions related to breast cancer micro-metastasis, indicating that the high-risk samples classified by 5-GPS are indeed those with occult metastases.

## Discussion

In this study, based on within-sample REOs, we developed a relapse-free prognostic signature, 5-GPS. We hypothesized that there exists micro-metastasis before surgery for those early-stage primary breast cancer patients who suffer relapse after surgical resection. Based on this hypothesis, we identified the signature from metastasis-related gene pairs consistently detected from samples measured by different microarray platforms. The signature can perform robustly for samples measured by the RNA-sequencing platform as well, demonstrating the unique advantage of the cross-platform robustness of the REO-based signature. The value of the signature was supported by the result that the RFS were significantly better in the predicted low-risk samples than in the predicted high-risk samples. Furthermore, there are clear genomic characteristics related to tumor metastasis in the high-risk samples. The majority rule used for 5-GPS may have insufficient sensitivity to identify metastases. We tried to reset a stricter criterion to identify non-metastatic patients: a patient is determined to be non-metastatic only if all gene pairs vote for low-risk and under this criterion the identified low-risk group has a better RFS and lower 5-year relapse rate than the high-risk group.

It is noteworthy that there was a significant number of patients whose cancers were relapsed within 5 years, but were classified as relapse-risk low by 5-GPS. This suggests an insufficient sensitivity of the 5-GPS signature combined with the majority rule to identify metastases. Considering a signature as an auxiliary tool for clinical decisions, it is reasonable to use a strict criterion to make conservative decisions on the identification of non-metastatic patients, while increasing the discovery sensitivity for metastatic patients. Therefore, a stricter criterion was applied to identify non-metastatic patients: a patient is classified as non-metastatic (low-risk) only if all gene pairs vote for the relapse-risk low and a patient is classified as metastatic (high-risk) only if all gene pairs vote for the relapse-risk high. Based on the criterion, for 544 samples integrated from GSE7390, GSE6532, GSE2034, and GSE4922 datasets, 23 patients were classified as low-risk, and 12 patients were classified as high-risk, where the low-risk group has a better RFS and a lower 5-year relapse rate than the high-risk group (HR = 13.71, 95%CI: 2.93–64.07, *p* = 1.64E-05, Supplementary Figure S7). The 5-year relapse rate in the low-risk group is 0, compared with 50% in the high-risk group. The relapse rate within 10 years is 4.17 and 66.67% in the low-risk group and high-risk group, respectively (Supplementary Figure S7A). Similar results were also obtained if one exception was allowed in the voting for the low-risk and high-risk groups (see Supplementary Figure S7B). This suggested that the 5-GPS signature is beneficial in reclassifying or identifying occult metastases.

Previously, we reported that the REO-based signatures are highly robust against experimental batch effects and differences in probe designs used in different platforms (Guan et al., 2016). The subtle quantitative gene expression values tend to be unreliable, the apparent disadvantage of qualitative nature of the relative orderings is in fact a unique advantage in terms of robustness (Guan et al., 2016). In this study, we constructed and validated the REO-based signature using microarray platforms from two companies and one RNA-sequencing platform. In comparison, the 70-gene signature assay approved by the FDA needs that tumor samples be sent to the Agendia Laboratories of the Netherlands Cancer Institute (Bueno-de-Mesquita et al., 2007). Therefore, the REO-based signatures are more convenient to apply under clinical settings than the signatures based on the quantitative expression values.

Although the REO-based signature constructed in this work demonstrated the cross-platform transferability, the number of significantly stable gene pairs was found to vary significantly among different platforms. For example, there are 1,442,839 significantly stable gene pairs selected from the samples measured by the Affymetrix’s microarray platform, while only 79,732 significantly stable gene pairs found in the samples measured by the Illumina’s microarray platform. The main effect of the number of gene pairs is the degree of difference between two groups, beside sample size and the number of measured genes. The magnitude of the difference was measured by differentially expressed genes (DEGs). Specially, 9557 DEGs (*T*-test, *p* < 0.01) were detected in GSE31448 but 1113 DEGs (*T*-test, *p* < 0.01) in EGAS00000000083, which suggested a small degree of difference between metastasis and non-metastasis groups and a large degree of uncertainty in sample composition associated to the Illumina dataset (EGAS00000000083). The weak and complex signals between metastasis and non-metastasis groups also results in low repeatability of REOs. Only 1690 gene pairs were shared and had consistent REO patterns between 1,442,839 gene pairs and 79,732gene pairs. The large discrepancy deserves further investigation.

In summary, the REO-based 5-GPS can aid the identification of early-stage breast cancer patients with micro-metastases who should receive adjuvant treatments.

## Data Availability Statement

All datasets presented in this study are included in the article/Supplementary Material.

## Author Contributions

ZG contributed conception and design of the study. QL, JZ, RC, and JL organized the database. NL, HC, KS, and YG performed the statistical analysis and interpretation of data. NL wrote the first draft of the manuscript. HC, XW, and ZG review and revision of the manuscript. All authors contributed to manuscript revision, read and approved the submitted version.

## Conflict of Interest

The authors declare that the research was conducted in the absence of any commercial or financial relationships that could be construed as a potential conflict of interest.

## Footnotes

1 http://www.ncbi.nlm.nih.gov/geo

2 http://www.ebi.ac.uk/ega//

3 http://cancergenome.nih.gov/

4 https://confluence.broadinstitute.org/display/GDAC/Download
