# Supplementary material for: A Five-Gene-Pair-Based Prognostic Signature for Predicting the Relapse Risk of Early Stage ER+ Breast Cancer
Source: Front Genet. 2020 Oct 29;11:566928. doi: 10.3389/fgene.2020.566928 (PMC7658391; doi:10.3389/fgene.2020.566928)
Supplement: Supplementary file 2 [file Table_2.DOCX]

**Table S2 Clinical characteristics of patients with ER+ breast cancer**

| Clinical characteristics | | Discovery cohort | | | | | | Validation cohort | |
| --- | --- | --- | --- | --- | --- | --- | --- | --- | --- |
|  |  | GSE19615 | GSE43365 | GSE31448 | EGAS00000000083 | GSE7390**^*^** | GSE6532**^*^** | GSE2034**^*^** | GSE4922**^*^** |
| All samples | | 40 | 58 | 108 | 283 | 134 | 85 | 209 | 116 |
|  | Median follow-up (months) | / | / | / | / | 125.8 (6.1-232) | 89.2 (6.1-176.8) | 86 (2-171) | 122 (6-152) |
| Age | Median age | 51.5 (32-84) | 57 (41-84) | 54 (24-84) | 61 (26-92) | 47 (24-60) | 55 (32-71) | / | 66 (32-90) |
|  | ≤55 | 23 (57.5%) | 27 (46.55%) | 56 (51.85%) | 80 (28.27%) | 115 (86%) | 44 (52%) | / | 34 (29%) |
|  | ＞55 | 17 (42.5%) | 31 (53.45%) | 52 (48.15%) | 203 (71.73%) | 19 (14*) | 41 (48%) | / | 82 (71%) |
| Tumor Grade | 1 | 13 (32.5%) | 17 (29.31%) | 30 (27.78%) | 2 (0.7%) | 29 (22%) | 29 (34%) | / | 48 (41%) |
|  | 2 | 12 (30%) | 32 (55.17%) | 46 (42.59%) | 98 (34.63%) | 68 (51%) | 62 (72%) | / | 58 (50%) |
|  | 3 | 15 (37.5%) | 9 (15.52%) | 32 (29.63%) | 99 (34.98%) | 35 (26%) | 12 (14%) | / | 10 (9%) |
|  | NA | / | / | / | 4 (1.41%) | 2 (1%) | 13 (15%) | / | 0 |
| Tumor Size | ≤2cm | 19 (47.5%) | 52 (89.66%) | / | 184 (65.02%) | 76 (57%) | 54 (64%) | / | 84 (72%) |
|  | ＞2cm | 21 (52.5%) | 6 (10.34%) | / | 99 (34.98%) | 58 (43%) | 31 (36%) | / | 32 (87%0 |
| Lymph Node | Negative | 21 (52.5%) | 6 (10.34%) | 78 (72.22%) | 99 (34.98%) | 134 (100%) | 85 (100%) | 209 (100%) | 116 (100%) |
|  | Positive | 19 (47.5%) | 52 (89.66%) | 30 (27.78%) | 184 (65.02%) | 0 | 0 | 0 | 0 |
| Tumor Stage | T1-T2 | / | / | / | / | 134 (100%) | 85 (100%) | 209 (100%) | 116 (100%) |
|  | T1 | / | / | 30 (27.78%) | 184 (65.02%) | / | / | / | / |
|  | T2-T4 | / | / | 78 (72.22%) | 99 (34.98%) | / | / | / | / |

**^*^** H. Cai, Z. Guo, et al. *Tamoxifen therapy benefit predictive signature coupled with prognostic signature of post-operative recurrent risk for early stage er+ breast cancer*, Oncotarget 6 (2015), no. 42, 44593-44608.
