# Supplementary material for: A Five-Gene-Pair-Based Prognostic Signature for Predicting the Relapse Risk of Early Stage ER+ Breast Cancer
Source: Front Genet. 2020 Oct 29;11:566928. doi: 10.3389/fgene.2020.566928 (PMC7658391; doi:10.3389/fgene.2020.566928)
Supplement: Supplementary file 7 [file Table_7.DOCX]

**Table S7 The predictive performance of the 5-GPS signature and 9-GPS**

|  | High-risk | Low-risk | Difference | | *p*-value | HR | 95%CI | High-risk ratio | C-index |
| --- | --- | --- | --- | --- | --- | --- | --- | --- | --- |
| **5-GPS** | | | | | | | | | |
| GSE7390  GSE6532 | 75 | 144 | | / | 1.11E-16 | 5.41 | 3.49-8.41 | 0.34 | 0.71 |
| GSE2034 | 112 | 97 | | / | 1.10E-02 | 1.79 | 1.14-2.83 | 0.546 | 0.58 |
| GSE4922 | 53 | 63 | | / | 3.56E-02 | 2.08 | 1.04-4.19 | 0.46 | 0.59 |
| **9-GPS** | | | | | | | | | |
| GSE7390 | 109 | 110 | | / | 1.02E-09 | 3.99 | 2.47-6.45 | 0.50 | 0.69 |
| GSE6532 |  |  |  |  |  |  |  |  |  |
| GSE2034 | 97 | 112 | | / | 2.71E-03 | 1.95 | 1.25-3.04 | 0.46 | 0.59 |
| GSE4922 | 34 | 82 | | / | 4.49E-03 | 2.61 | 1.31-5.19 | 0.29 | 0.60 |
| **5-GPS+9-GPS** | | | | | | | | | |
| GSE7390 | 61 | 96 | | 62 | <2E-16 | 3.13 | 2.35-4.16 | 0.28 | 0.76 |
| GSE6532 |  |  |  |  |  |  |  |  |  |
| GSE2034 | 63 | 73 | | 63 | 0.001 | 1.56 | 1.19-2.04 | 0.30 | 0.61 |
| GSE4922 | 21 | 50 | | 45 | 0.002 | 2.03 | 1.29-3.18 | 0.18 | 0.64 |
