# Supplementary material for: A Five-Gene-Pair-Based Prognostic Signature for Predicting the Relapse Risk of Early Stage ER+ Breast Cancer
Source: Front Genet. 2020 Oct 29;11:566928. doi: 10.3389/fgene.2020.566928 (PMC7658391; doi:10.3389/fgene.2020.566928)
Supplement: Supplementary file 8 [file Table_8.DOCX]

**Table S8 Significantly different copy region frequencies between high- and low-risk samples（FDR <0.05）**

| Copy region | Variation ratio of high-risk group | Variation ratio of low-risk group | Difference | Statistical significance | |
| --- | --- | --- | --- | --- | --- |
|  |  |  |  | p_value | FDR |
| Deletion_Peak__1 | 0.6277 | 0.2262 | 0.4014 | 2.75E-11 | 1.93E-09 |
| Amplification_Peak_18 | 0.5000 | 0.1629 | 0.3371 | 1.83E-09 | 6.41E-08 |
| Deletion_Peak_16 | 0.5532 | 0.2081 | 0.3450 | 4.71E-09 | 1.10E-07 |
| Amplification_Peak__7 | 0.4255 | 0.1222 | 0.3034 | 8.24E-09 | 1.44E-07 |
| Amplification_Peak_17 | 0.3830 | 0.1041 | 0.2789 | 2.91E-08 | 3.40E-07 |
| Deletion_Peak_17 | 0.5426 | 0.2127 | 0.3299 | 2.50E-08 | 3.40E-07 |
| Amplification_Peak_24 | 0.5957 | 0.2624 | 0.3333 | 4.35E-08 | 4.35E-07 |
| Deletion_Peak_21 | 0.4149 | 0.1267 | 0.2882 | 5.10E-08 | 4.46E-07 |
| Deletion_Peak__2 | 0.5532 | 0.2353 | 0.3179 | 9.86E-08 | 7.67E-07 |
| Deletion_Peak_25 | 0.6064 | 0.2896 | 0.3168 | 2.79E-07 | 1.96E-06 |
| Deletion_Peak_35 | 0.4468 | 0.1674 | 0.2794 | 4.29E-07 | 2.73E-06 |
| Amplification_Peak_11 | 0.7660 | 0.4706 | 0.2954 | 1.10E-06 | 5.48E-06 |
| Amplification_Peak_28 | 0.6596 | 0.3575 | 0.3021 | 1.08E-06 | 5.48E-06 |
| Deletion_Peak_24 | 0.6489 | 0.3484 | 0.3005 | 1.08E-06 | 5.48E-06 |
| Deletion_Peak_20 | 0.4255 | 0.1629 | 0.2626 | 1.47E-06 | 6.86E-06 |
| Deletion_Peak_34 | 0.7340 | 0.4480 | 0.2861 | 4.00E-06 | 1.75E-05 |
| Deletion_Peak__3 | 0.4043 | 0.1674 | 0.2368 | 1.23E-05 | 5.06E-05 |
| Deletion_Peak_36 | 0.4468 | 0.2036 | 0.2432 | 1.73E-05 | 6.75E-05 |
| Deletion_Peak_18 | 0.3723 | 0.1493 | 0.2230 | 2.23E-05 | 8.22E-05 |
| Deletion_Peak_37 | 0.4149 | 0.1810 | 0.2339 | 2.89E-05 | 9.75E-05 |
| Deletion_Peak_40 | 0.3298 | 0.1222 | 0.2076 | 2.92E-05 | 9.75E-05 |
| Amplification_Peak_20 | 0.3511 | 0.1448 | 0.2063 | 6.58E-05 | 2.09E-04 |
| Deletion_Peak_19 | 0.3298 | 0.1312 | 0.1986 | 7.67E-05 | 2.34E-04 |
| Amplification_Peak_12 | 0.2979 | 0.1086 | 0.1893 | 9.57E-05 | 2.68E-04 |
| Deletion_Peak_29 | 0.5638 | 0.3258 | 0.2380 | 9.49E-05 | 2.68E-04 |
| Amplification_Peak_15 | 0.4787 | 0.2579 | 0.2208 | 2.07E-04 | 5.57E-04 |
| Amplification_Peak_23 | 0.4149 | 0.2127 | 0.2022 | 3.22E-04 | 8.34E-04 |
| Amplification_Peak__3 | 0.3191 | 0.1403 | 0.1789 | 5.08E-04 | 1.27E-03 |
| Deletion_Peak__5 | 0.3830 | 0.1900 | 0.1929 | 5.44E-04 | 1.27E-03 |
| Deletion_Peak_38 | 0.2447 | 0.0905 | 0.1542 | 5.31E-04 | 1.27E-03 |
| Deletion_Peak_22 | 0.3191 | 0.1493 | 0.1698 | 1.08E-03 | 2.43E-03 |
| Deletion_Peak_30 | 0.3191 | 0.1538 | 0.1653 | 1.26E-03 | 2.75E-03 |
| Amplification_Peak__9 | 0.5426 | 0.3439 | 0.1987 | 1.62E-03 | 3.43E-03 |
| Amplification_Peak_21 | 0.2340 | 0.0995 | 0.1345 | 2.44E-03 | 5.03E-03 |
| Deletion_Peak_32 | 0.3617 | 0.1991 | 0.1626 | 2.80E-03 | 5.59E-03 |
| Amplification_Peak__1 | 0.8511 | 0.6923 | 0.1588 | 3.15E-03 | 6.12E-03 |
| Deletion_Peak_27 | 0.2021 | 0.0814 | 0.1207 | 3.76E-03 | 7.12E-03 |
| Deletion_Peak__4 | 0.2872 | 0.1403 | 0.1470 | 3.87E-03 | 7.13E-03 |
| Deletion_Peak_39 | 0.1702 | 0.0588 | 0.1114 | 4.60E-03 | 8.25E-03 |
| Deletion_Peak_14 | 0.5426 | 0.3710 | 0.1715 | 6.02E-03 | 1.05E-02 |
| Deletion_Peak_23 | 0.2872 | 0.1493 | 0.1379 | 7.21E-03 | 1.23E-02 |
| Deletion_Peak_31 | 0.3085 | 0.1719 | 0.1366 | 1.01E-02 | 1.68E-02 |
| Deletion_Peak_12 | 0.1489 | 0.0543 | 0.0946 | 1.20E-02 | 1.92E-02 |
| Deletion_Peak_13 | 0.2447 | 0.1267 | 0.1180 | 1.21E-02 | 1.92E-02 |
| Amplification_Peak__4 | 0.3404 | 0.2036 | 0.1368 | 1.44E-02 | 2.14E-02 |
| Amplification_Peak__5 | 0.1915 | 0.0905 | 0.1010 | 1.45E-02 | 2.14E-02 |
| Amplification_Peak__6 | 0.4681 | 0.3167 | 0.1513 | 1.46E-02 | 2.14E-02 |
| Deletion_Peak_11 | 0.4574 | 0.3077 | 0.1498 | 1.41E-02 | 2.14E-02 |
| Amplification_Peak_22 | 0.2340 | 0.1222 | 0.1119 | 1.69E-02 | 2.41E-02 |
| Amplification_Peak_10 | 0.4681 | 0.3258 | 0.1423 | 2.14E-02 | 2.99E-02 |
| Deletion_Peak__6 | 0.3511 | 0.2217 | 0.1293 | 2.42E-02 | 3.33E-02 |
| Deletion_Peak_10 | 0.4362 | 0.3032 | 0.1330 | 2.74E-02 | 3.69E-02 |
| Deletion_Peak__7 | 0.2021 | 0.1041 | 0.0981 | 2.85E-02 | 3.77E-02 |
| Deletion_Peak_33 | 0.6596 | 0.7783 | -0.119 | 3.44E-02 | 4.45E-02 |
| Amplification_Peak__2 | 0.8191 | 0.7059 | 0.1133 | 3.65E-02 | 4.64E-02 |
