# Supplementary material for: A Five-Gene-Pair-Based Prognostic Signature for Predicting the Relapse Risk of Early Stage ER+ Breast Cancer
Source: Front Genet. 2020 Oct 29;11:566928. doi: 10.3389/fgene.2020.566928 (PMC7658391; doi:10.3389/fgene.2020.566928)
Supplement: Supplementary file 9 [file Table_9.DOCX]

**Table S9 Significantly different mutation frequencies between stage III-IV high-risk samples and stage I-II low-risk samples**

| Low-risk group VS high-risk group  （*p* <0.05） | The number of high-risk group | The number of low-risk group | Mutation frequencies of high-risk group | Mutation frequencies of low-risk group | Difference |
| --- | --- | --- | --- | --- | --- |
| **TP53** | 77 | 194 | 0.272727 | 0.128866 | 0.143861 |
| **FAT3** | 77 | 194 | 0.064935 | 0.015464 | 0.049471 |
| **HMCN1** | 77 | 194 | 0.064935 | 0.015464 | 0.049471 |
| **ITPR3** | 77 | 194 | 0.051948 | 0.005155 | 0.046793 |
| ADAMTS9 | 77 | 194 | 0.038961 | 0 | 0.038961 |
| DENND1B | 77 | 194 | 0.038961 | 0 | 0.038961 |
| F5 | 77 | 194 | 0.038961 | 0 | 0.038961 |
| F8 | 77 | 194 | 0.038961 | 0 | 0.038961 |
| FAM184A | 77 | 194 | 0.038961 | 0 | 0.038961 |
| MLLT10 | 77 | 194 | 0.038961 | 0 | 0.038961 |
| MOV10L1 | 77 | 194 | 0.038961 | 0 | 0.038961 |
| NRXN3 | 77 | 194 | 0.038961 | 0 | 0.038961 |
| OR5H1 | 77 | 194 | 0.038961 | 0 | 0.038961 |
| TNKS1BP1 | 77 | 194 | 0.038961 | 0 | 0.038961 |
| **PIK3CA** | 77 | 194 | 0.233766 | 0.42268 | -0.18891 |
