# Supplementary material for: A Five-Gene-Pair-Based Prognostic Signature for Predicting the Relapse Risk of Early Stage ER+ Breast Cancer
Source: Front Genet. 2020 Oct 29;11:566928. doi: 10.3389/fgene.2020.566928 (PMC7658391; doi:10.3389/fgene.2020.566928)
Supplement: Supplementary file 10 [file Table_10.DOCX]

**Supplementary Materials**

**Figure S1. The Kaplan-Meier curves of RFS for integrated data by 5-GPS.**

**Figure S2. The Clinical Information of High-risk and Low-risk Group in Discovery and Validation Cohort**

**Figure S3. The proportion of the high-risk samples in each stage with different tumor cell percent using 5-GPS.** A. the samples with tumor cell percent under 50%. B. the samples with tumor cell percent upper 50%. C. the samples with tumor cell percent under 60%. D. the samples with tumor cell percent upper 60%.

**Figure S4. The Comparison of 5-GPS and 9-GPS.** There are 544 samples including GSE7390, GSE6532, GSE2034 and GSE4922 cohort.

**Figure S5. The proportion of the high-risk samples in each stage using 9-GPS.**

**Figure S6. The predictive performance of hybrid model of 5-GPS and 9-GPS.** The Kaplan-Meier curves of RFS for the early-stage breast cancer patients in (A, B, C) the data cohorts. The three curves are low-risk group, difference group and high-risk group.

**Figure S7. The performance of the 5-GPS signature.** The Kaplan-Meier curves of RFS for all the breast cancer patients using strict voting criterion of 5-GPS(A). The Kaplan-Meier curves of RFS for all the breast cancer patients using one exception was allowed in the voting for the low-risk or high-risk groups(B)

**Figure S8. The ROC curves for 5-GPS.** Time point of the ROC curve was set as 120 months.
