# Supplementary material for: A Five-Gene-Pair-Based Prognostic Signature for Predicting the Relapse Risk of Early Stage ER+ Breast Cancer
Source: Front Genet. 2020 Oct 29;11:566928. doi: 10.3389/fgene.2020.566928 (PMC7658391; doi:10.3389/fgene.2020.566928)
Supplement: Supplementary file 11 [file Table_11.DOCX]

##Input,control_exp,case_exp,gneid

source("All_Pair.r")

source("Pair_Compare.r")

control_allpair<-All_Pair(control_exp,geneid)

save(control_allpair,file="control_allpair.RData")

rm(control_allpair)

gc()

case_allpair<-All_Pair(case_exp,geneid)

save(case_allpair,file="case_allpair.RData")

rm(case_exp,control_exp)

load("control_allpair.RData")

fdr1<-p.adjust(control_allpair[,3],'BH',length(control_allpair[,3]))

load("case_allpair.RData")

fdr2<-p.adjust(case_allpair[,3],'BH',length(case_allpair[,3]))

cut_off<-0.05

controlP<-control_allpair[fdr1<cut_off,1:2]

caseP<-case_allpair[fdr2<cut_off,1:2]

caseP<-cbind(as.integer(caseP[,1]),as.integer(caseP[,2]))

controlP<-cbind(as.integer(controlP[,1]),as.integer(controlP[,2]))

rm(control_allpair,case_allpair)

gc()

pair_out<-Pair_Compare(controlP,caseP,geneid)

consis_loc<-pair_out$consis_loc

rever_loc<-pair_out$rever_loc

rm(caseP,controlP,pair_out)

All_Pair<-function(control_exp,geneid){

control_exp<-as.matrix(control_exp)

geneid<-as.matrix(geneid)

len<-length(geneid)

all_pair<-matrix(NA,choose(len,2),4)

m<-ncol(control_exp)

j=1

for (i in 1:(len-1)){

exp_temp<-control_exp[i,]

relative<-matrix(rep(exp_temp,(len-i)),ncol=m,byrow=T)-control_exp[(1+i):len,]

relative1<-relative>0

relative2<-relative<0

sum_temp1<-as.matrix(rowSums(relative1))

sum_temp2<-as.matrix(rowSums(relative2))

rm(exp_temp,relative1,relative2)

gc()

ratio1<-as.matrix(sum_temp1/m)

ratio2<-as.matrix(sum_temp2/m)

index1<-as.matrix(ratio1>0.5)

index2<-as.matrix(ratio2>0.5)

pair<-matrix(NA,len-i,2)

pair[,1]<-geneid[i]

pair[,2]<-geneid[(i+1):len]

sum1<-sum_temp1[index1,]

p1<-1-pbinom(sum1-1,m,0.5)

if (length(p1)==1){

pair11<-rbind(as.matrix(pair[index1,c(1,2)]),p1,as.matrix(ratio1[index1,1]))

pair1=t(pair11)} else

{pair1=cbind(pair[index1,c(1,2)],p1,ratio1[index1,1])}

sum2<-sum_temp2[index2,]

p2<-1-pbinom(sum2-1,m,0.5)

if (length(p2)==1){

pair22<-rbind(as.matrix(pair[index2,c(2,1)]),p2,as.matrix(ratio2[index2,1]))

pair2=t(pair22)} else

{pair2<-cbind(pair[index2,c(2,1)],p2,ratio2[index2,1])}

x1<-dim(pair1)[1]

x2<-dim(pair2)[1]

xx<-x1+x2

all_pair[j:(j+xx-1),1:4]<-rbind(pair1,pair2)

j<-j+xx

rm(sum_temp1,sum_temp2,index1,index2,pair,sum1,p1,pair1,sum2,p2,pair2,ratio1,ratio2,xx)}

ind<-is.na(all_pair[,1])

all_pair<-all_pair[!ind,]

rm(ind)

gc()

return(all_pair)

}

Pair_Compare<-function(controlP,caseP,gid){

loc_c1<-match(controlP[,1],gid)

loc_c2<-match(controlP[,2],gid)

loc_t1<-match(caseP[,1],gid)

loc_t2<-match(caseP[,2],gid)

aa<-length(gid)

n1<-length(loc_c1)

n2<-length(loc_t1)

cont_mat<-matrix(0,aa,aa)

locc<-cbind(row=loc_c1,col=loc_c2)##row>col

cont_mat[locc]<-1

rm(locc,loc_c1,loc_c2)

case_mat<-matrix(0,aa,aa)

locc<-cbind(row=loc_t1,col=loc_t2)

case_mat[locc]<-1

rm(locc,loc_t1,loc_t2)

gc()

consis<-cont_mat+case_mat

reverse<-cont_mat+t(case_mat)

rm(cont_mat,case_mat,aa)

gc()

consis_loc<-which(consis==2,arr.ind = T)##

m<-dim(consis_loc)[1]

rever_loc<-which(reverse==2,arr.ind = T)##normal:row>col;case:row<col

n<-dim(rever_loc)[1]

ratio<-m/(m+n)

p<-1-pbinom(m-1,m+n,0.5)

result<-c(n1,n2,m,m+n,ratio,p,m/n1,m/n2)

output<-list(result=result,consis_loc=consis_loc,rever_loc=rever_loc)

}
