# Supplementary figures and images for: A Five-Gene-Pair-Based Prognostic Signature for Predicting the Relapse Risk of Early Stage ER+ Breast Cancer
Source: Front Genet. 2020 Oct 29;11:566928. doi: 10.3389/fgene.2020.566928 (PMC7658391; doi:10.3389/fgene.2020.566928)

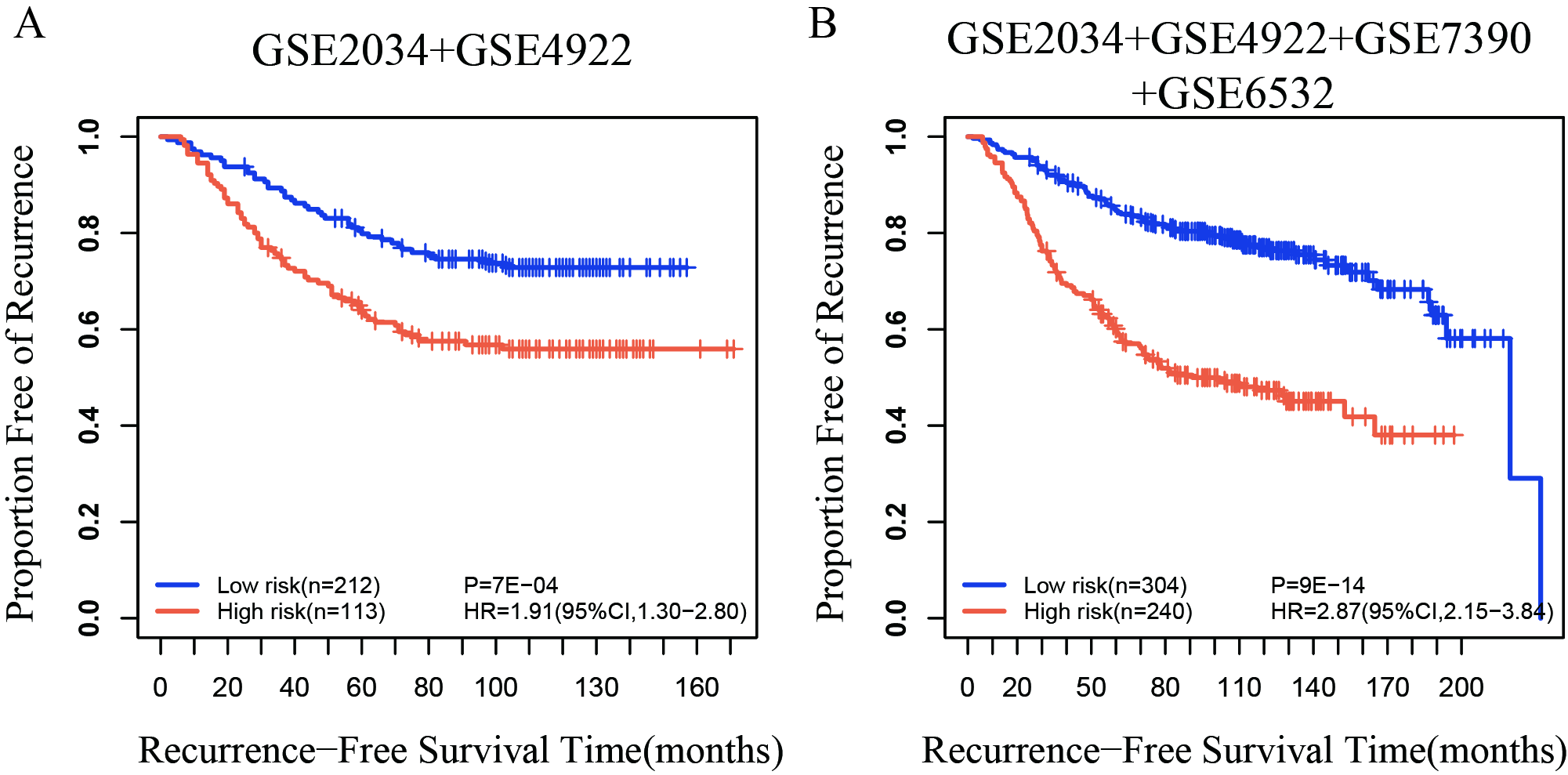

Supplement: Supplementary file 12 [file Image_1.TIF]

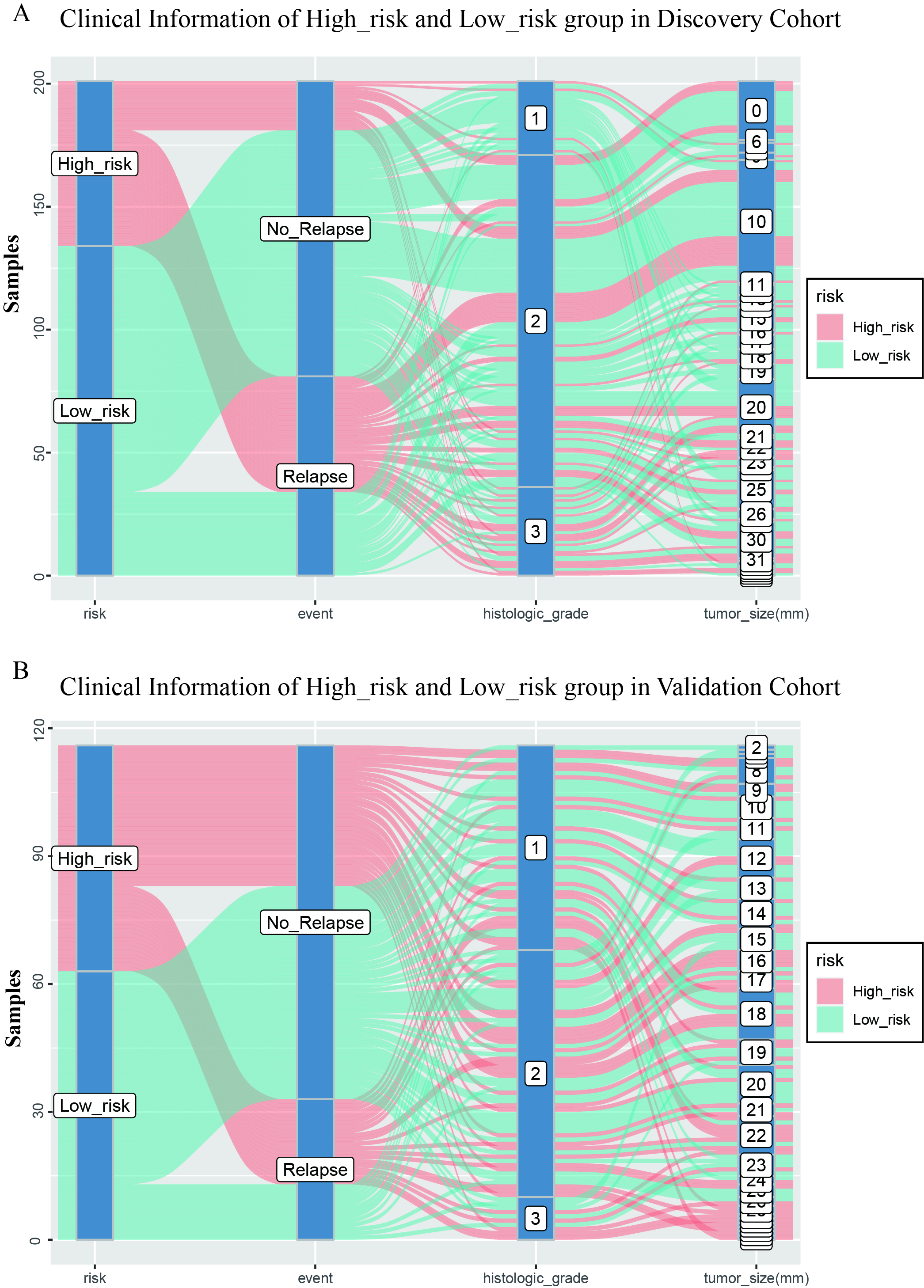

Supplement: Supplementary file 13 [file Image_2.TIF]

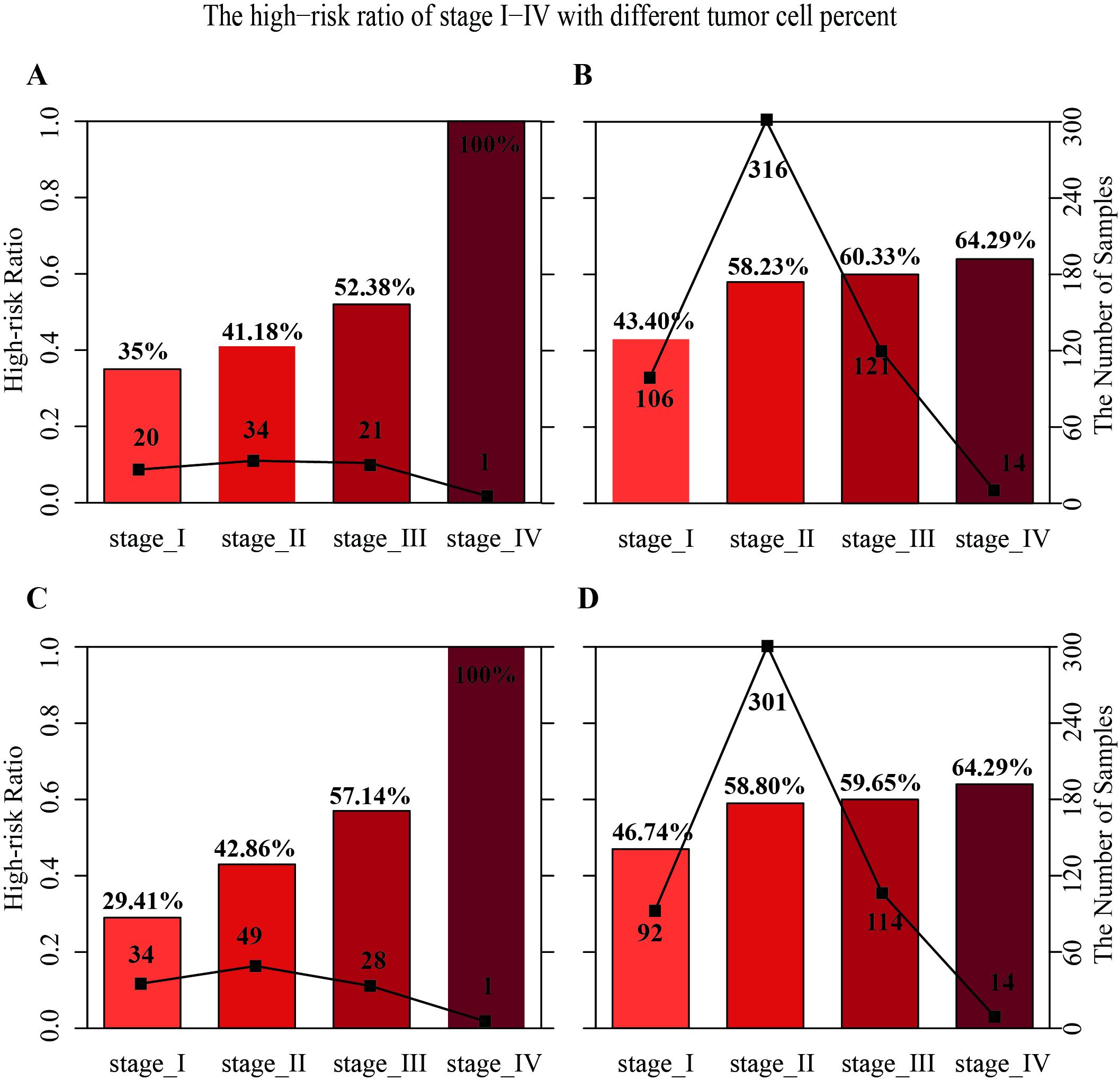

Supplement: Supplementary file 14 [file Image_3.TIF]

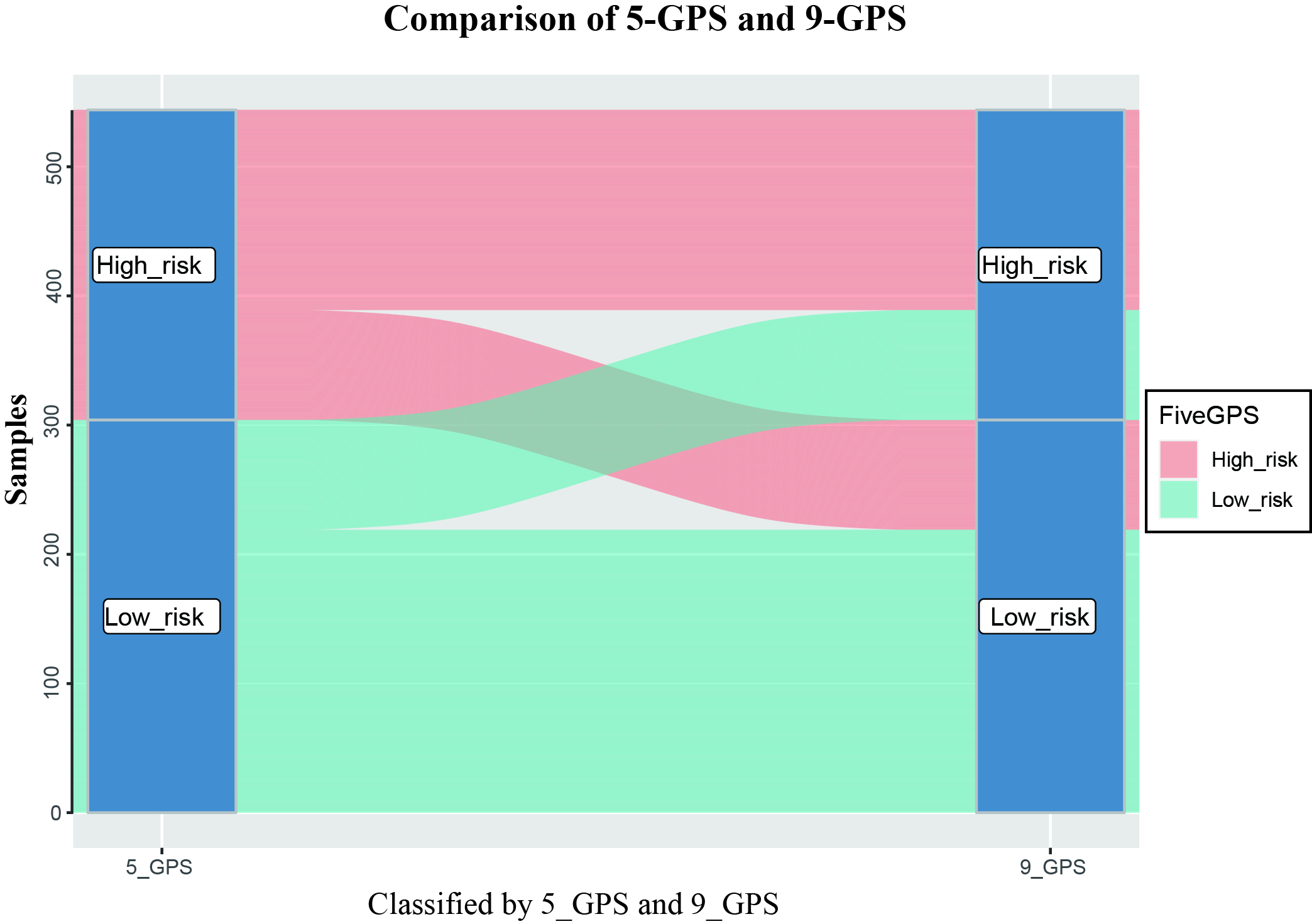

Supplement: Supplementary file 15 [file Image_4.TIF]

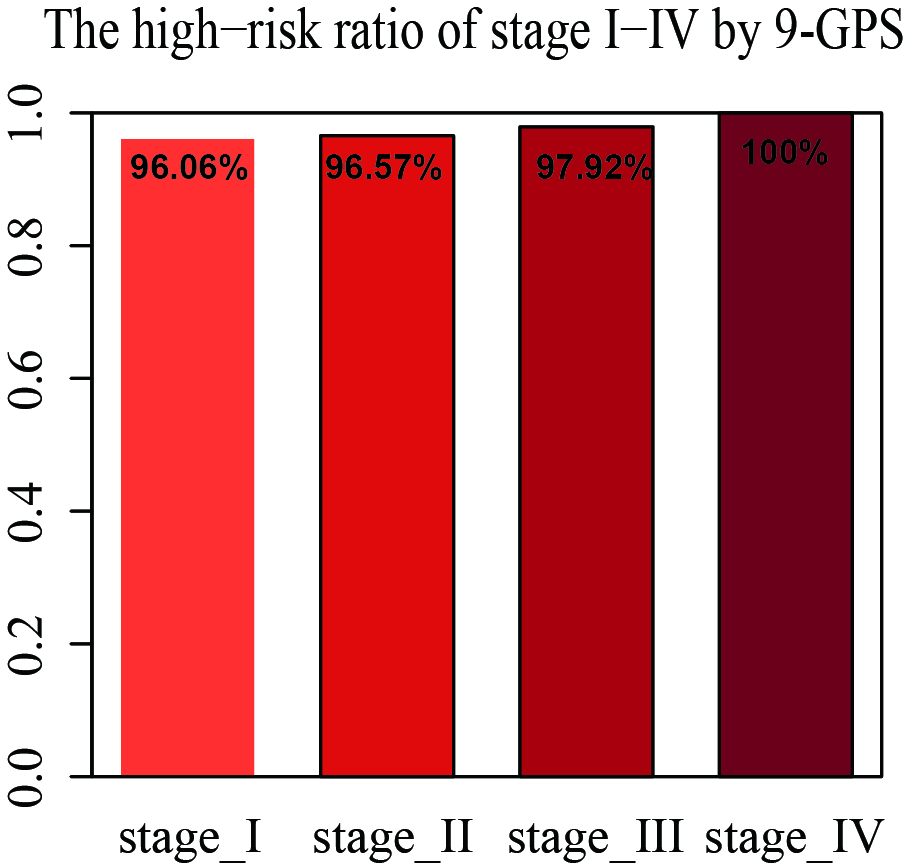

Supplement: Supplementary file 16 [file Image_5.TIF]

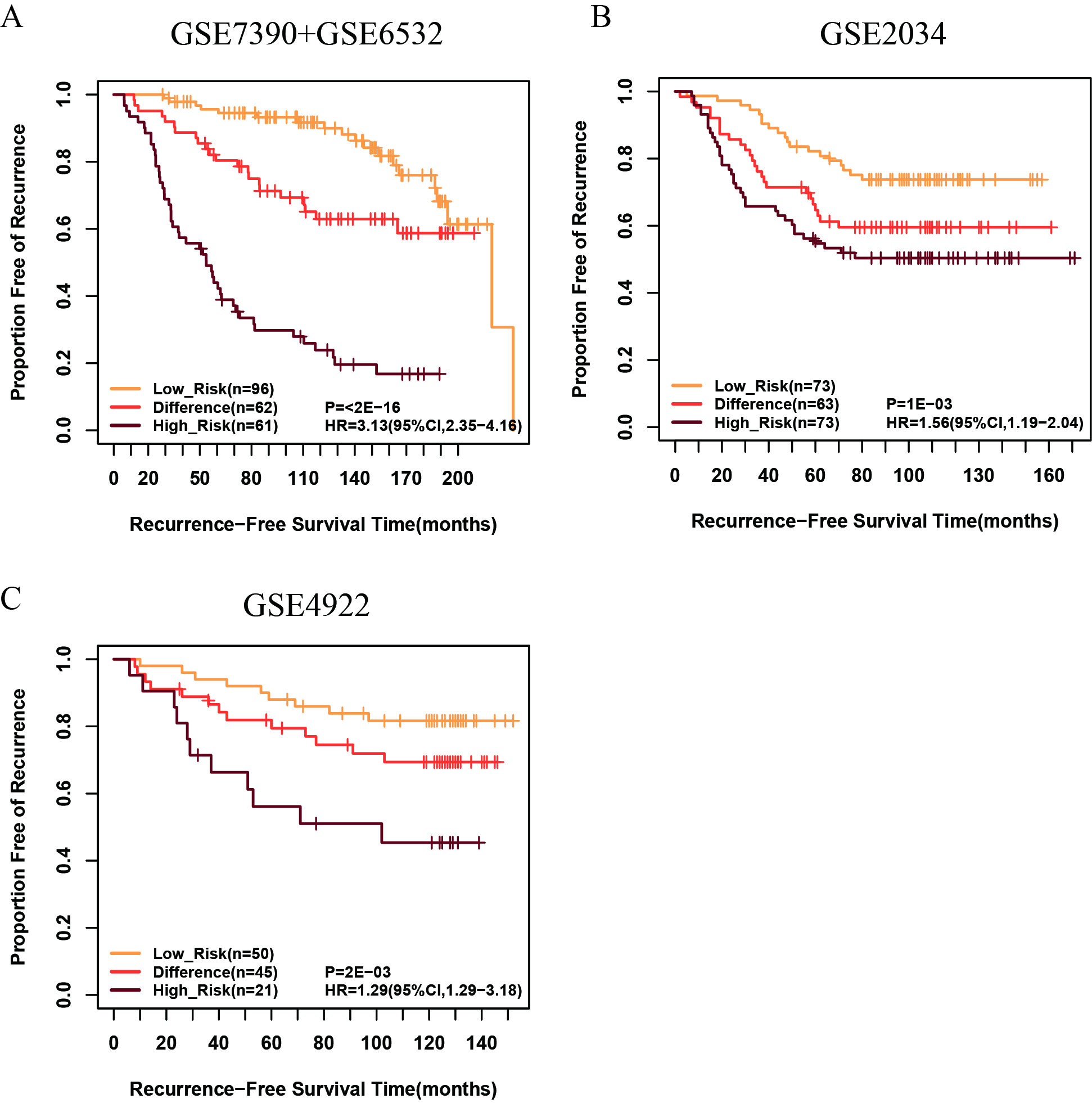

Supplement: Supplementary file 17 [file Image_6.TIF]

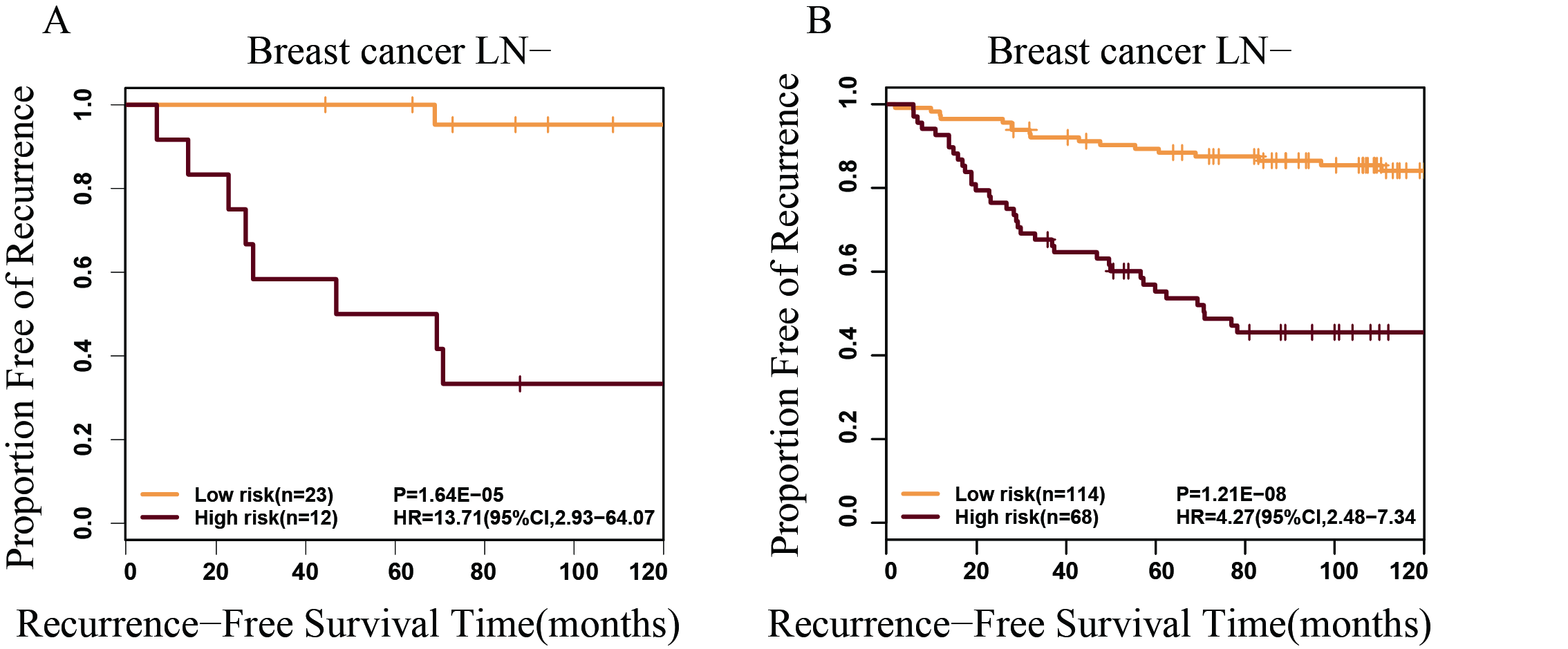

Supplement: Supplementary file 18 [file Image_7.TIF]

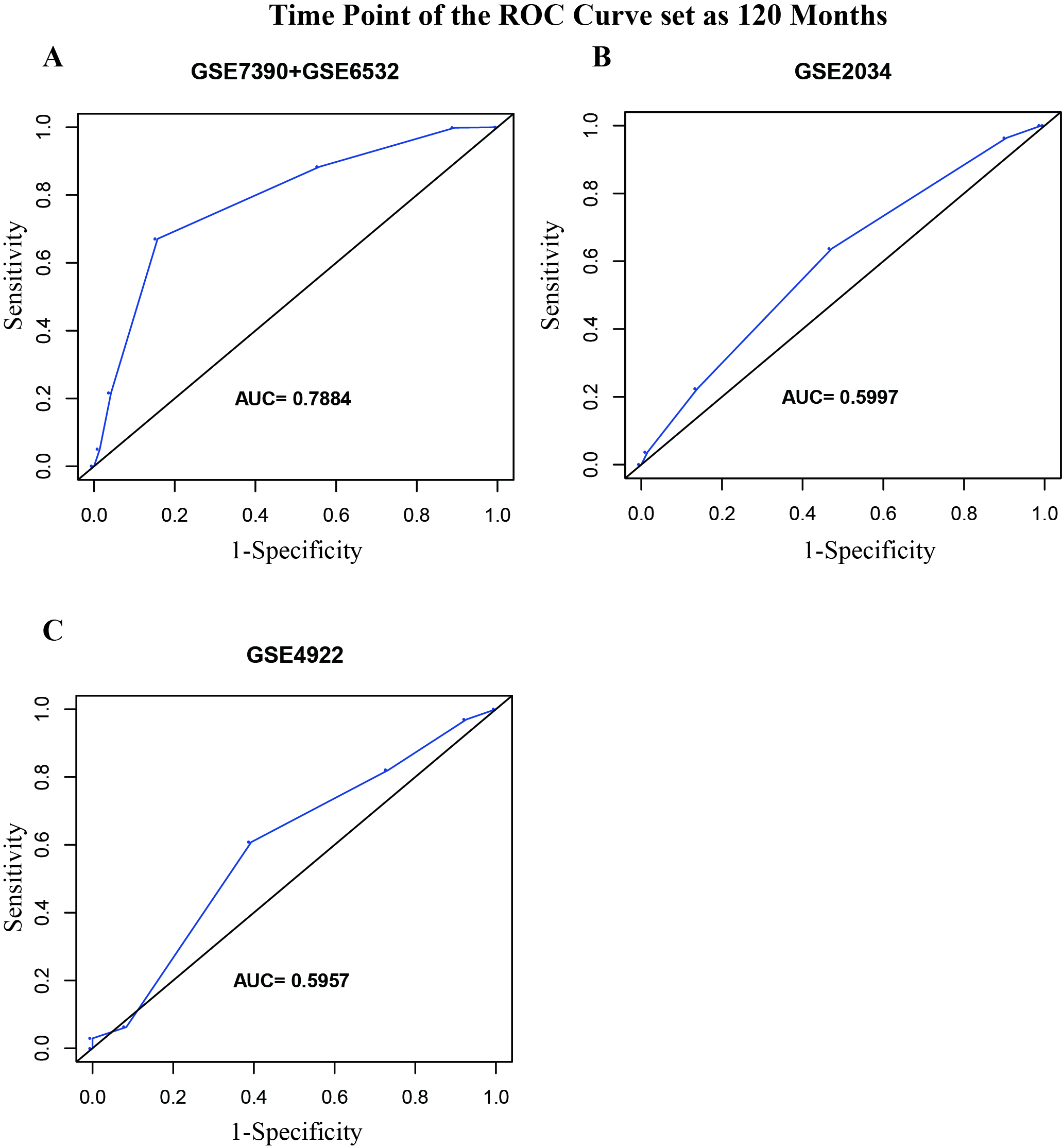

Supplement: Supplementary file 19 [file Image_8.TIF]
